# The dangers of lithotomy positioning in the operating room: case report of bilateral lower extremity compartment syndrome after a 90-minutes surgical procedure

**DOI:** 10.1186/s13037-016-0106-9

**Published:** 2016-07-26

**Authors:** Nicole Stornelli, Frank B. Wydra, Justin J. Mitchell, Philip F. Stahel, Stefka Fabbri

**Affiliations:** 1 Department of Obstetrics and Gynecology, Denver Health Medical Center and University of Colorado School of Medicine, Denver, CO 80204 USA; 2 Department of Orthopaedic Surgery, Denver Health Medical Center and University of Colorado School of Medicine, Denver, CO 80204 USA

**Keywords:** Compartment syndrome, Fasciotomy, Lithotomy position, Surgical complication, Adverse event, Patient safety

## Abstract

**Background:**

Lower extremity acute compartment syndrome after gynecologic surgery in the lithotomy position is a rare, yet potentially devastating complication. A high level of suspicion is paramount for early recognition and mitigation of acute compartment syndrome originating from prolonged surgery in lithotomy position.

**Case presentation:**

A 23-year-old female, gravida 1, para 0, underwent a laparoscopic salpingectomy for a ruptured ectopic pregnancy. Surgical time was 90 min. Postoperatively, the patient developed acute compartment syndrome of both legs necessitating emergent bilateral four-compartment fasciotomies, with repeated returns to the operating room for 2nd look procedures and delayed wound closures. The patient regained full function within 3 months and returned to an unrestricted baseline activity level.

**Conclusion:**

Technical diligence in applying a lithotomy position is paramount for preventing postoperative lower extremity compartment syndrome. A high level of suspicion for this severe complication in conjunction with early recognition and immediate surgical management can mitigate long-term adverse sequelae and improve postoperative outcomes.

## Background

Acute compartment syndrome (ACS) of the lower extremities is a known complication after surgical positioning in lithotomy or hemilithotomy position [1–3]. As most gynecological procedures require a lithotomy position by default, these patients are at particular risk for developing postoperative compartment syndrome, particularly after prolonged surgical procedures lasting beyond 2–4 h [4, 5]. Although a rare complication, delayed diagnosis of ACS can lead to irreversible muscle necrosis with the potential for debilitating lower extremity dysfunction and a high risk for requiring delayed lower limb amputations [6, 7]. Thus, prompt recognition and surgical intervention are paramount for mitigating this severe postoperative complication [8–10]. The large bulk of the existing literature on ACS secondary to lithotomy positioning are case reports either of prolonged surgical procedures beyond 4 h, or restricted to unilateral well-leg compartment syndrome [11, 12]. In this report, we describe the rare case of a young 23-year old patient who developed bilateral lower extremity ACS following a routine 90-min laparoscopic salpingectomy in lithotomy position for an ectopic pregnancy. Tips and tricks on how to prevent and mitigate this severe intraoperative complication are discussed.

## Case presentation

**Fig. 1:**
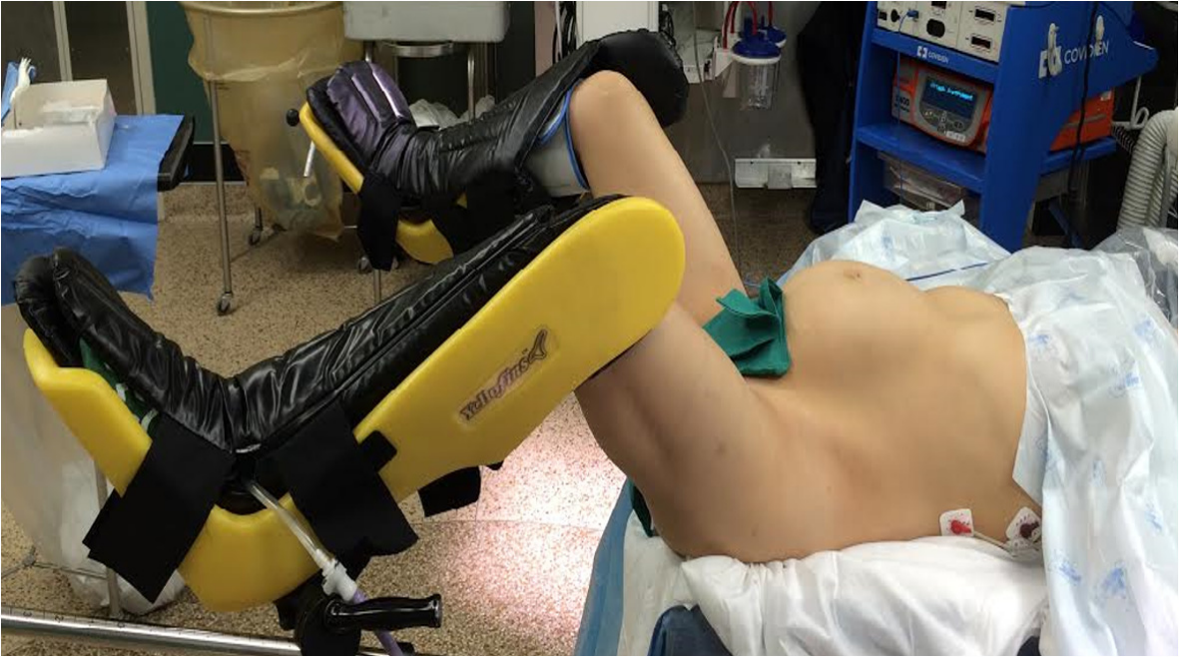
Demonstration of lithotomy position technique applied at the authors’ institution

A 23-year-old woman, gravida 1, para 0, at unknown gestational age presented to our emergency department with lower abdominal pain, vaginal spotting, and lightheadedness for 2 days. The patient’s medical history was significant for morbid obesity with body mass index, history of Chlamydia infection and pelvic inflammatory disease at age 15, and laparoscopic cholecystectomy at age 19. Physical examination including a transvaginal ultrasonographic evaluation revealed a ruptured ectopic pregnancy. The patient was hemodynamically stable and underwent a laparoscopic right salpingectomy. The procedure was performed in a standard “low” lithotomy position (using Allen® stirrups). Care was taken not to flex hips or knees beyond 90°, with hip abduction less than 45° and neutral hip rotation (Fig. 1). The patient was in Trendelenburg position to allow for adequate visualization of the pelvis. Pneumatic compression devices on both calves were in place throughout the procedure. Skin-to-skin surgical time was 90 min due to the need for lysis of omental adhesion and the presence of pelvic adhesive disease consistent with the patient’s prior surgical and gynecologic history. Intraoperative findings and pathologic evaluation confirmed the diagnosis of a ruptured ectopic pregnancy. Immediately upon awakening from general anesthesia, the patient complained of severe bilateral calf pain. Initial evaluation of the lower extremities revealed no compression marks, ecchymoses, erythema or edema, and the peripheral neurovascular exam was unremarkable. Serum electrolytes were within normal limits. Serum creatine kinase (CK) was elevated at 22,760 units/L (Norm: 38–176 units/L), consistent with rhabdomyolysis. An urgent bilateral lower extremity Doppler ultrasound was obtained which ruled out a deep venous thrombosis. The patient was treated symptomatically with analgesics and muscle relaxants. As her pain continued to escalate, a concern for acute compartment syndrome (ACS) was raised and the orthopedic surgery service was consulted. Based on the high level of suspicion for ACS in light of the clinical exam and exacerbating pain out of proportion, the patient was taken back emergently to the operating room by the orthopedic surgery team for bilateral lower extremity four-compartment fasciotomies. Surgery was accomplished approximately 8 h after her initial laparoscopic surgery. Intraoperatively, the diagnosis of ACS was confirmed. All muscles in the four compartments in bilateral legs were viable on clinical exam and bovie stimulation testing, without any signs of muscle necrosis. The patient required two additional staged surgical procedures for scheduled 2nd looks, soft tissue debridement and delayed primary wound closures. Her serum CK decreased to 1278 units/L prior to discharge and she did not sustain any renal failure or crush syndrome throughout the hospitalisation. The patient’s serum creatinine remained in a normal range of 0.6–0.8 mg/dL (Norm: 0.5–1.0 mg/dL). She was discharged home on postoperative day four after definitive wound closure, with home physical therapy arranged. She had an uneventful further recovery, with pristine wound healing, no infection and a normal neurovascular status on bilateral lower extremities on follow-up exam. By 3 months after discharge from the hospital, she had regained full unrestricted function and a normal quality of life.

### Discussion

We present a rare case of bilateral lower extremity acute compartment syndrome that developed in a healthy 23-year-old female after undergoing a laparoscopic salpingectomy for ruptured ectopic pregnancy in the usual lithotomy position. This case highlights a rare complication that has been previously associated with lithotomy or hemilithotomy positioning [2, 5, 12, 13]. Lower extremity ACS is a pathologic condition in which increased tissue pressure within a closed osseofascial space compromises blood circulation and normal function of tissues within the compartment leading to tissue hypoxia and necrosis [8, 14]. If left untreated, patients with ACS can develop muscle contracture, sensory deficits, paralysis, possible need for limb amputation, and potentially multi-system organ failure secondary to crush syndrome [15, 16]. The osseofascial lining of the four lower extremity compartments (anterior, lateral, superficial and deep posterior) form an enclosed environment of muscle, blood vessels and nerves with limited ability to expand or accommodate increased volume or pressure [8, 17]. ACS in the postoperative state occurs as a result of intraoperative compression with a prolonged decrease in arterial pressure and/or increase in venous pressure, followed by reperfusion [18]. Local ischemia can subsequently damage endothelial cells resulting in leakage of proteins and fluid into the interstitial space. The subsequent increase in interstitial pressure elevates compartmental pressures, thus perpetuating the cycle of hypoperfusion and tissue ischemia [19]. Specifically, in the lithotomy position, ischemia occurs from compressive forces of the external leg holsters and diminished blood flow from leg elevation and kinking of the popliteal artery, leading to ischemia/reperfusion injury with subsequent compartment syndrome [1]. When the lower extremities are taken out of lithotomy position and elevation, limb reperfusion occurs and can lead to injury with the formation of oxygen free radicals and cytokines that perpetuate endothelial damage and interstitial edema [20]. In addition to lithotomy position, other risk factors associated with ACS after surgery include: ankle dorsiflexion, Trendelenburg position, leg holders, length of surgery greater than 2 h, intraoperative hypotension or hypovolemia, and epidural analgesia [1]. Bauer and colleagues recently published two articles documenting cases of lower extremity ACS following gynecologic surgery in the lithotomy position and found that the vast majority of ACS cases occurred following procedures lasting longer than 4 h [4, 12]. Other studies have demonstrated that calf compartment pressure increases at a rate of 1.1 mmHg per hour in the lithotomy position [1]. In addition, elevation of the leg above heart level (“high” lithotomy positon) has been associated with increased risk of compartment syndrome [21]. Intermittent pneumatic compressive devices used for the prevention of deep venous thrombosis intraoperatively, as applied in the patient presented in this case report, have also been identified as causative factors for ACS [22]. We postulate that lithotomy with adjunctive Trendelenburg position was likely the contributory root cause of bilateral lower extremity ACS in the young 23-old patient described in this case report. Multiple technical tricks have been described to prevent ACS in lithotomy position, including intraoperative repositioning of the legs, avoiding pressure in the popliteal fossa and kinking of the popliteal artery by avoiding knee flexion beyond 90° [1, 23]. Lower extremity ACS remains a clinical diagnosis, and immediate recognition and surgical management are paramount in avoiding long-term impairment and poor outcomes [14]. The most common clinical symptom is represented by uncontrolled pain out of proportion and exacerbated pain by passive stretch of the toes and ankle joint [14]. Measurement of the compartment pressure can aid in the diagnosis, albeit there is dispute among orthopedic surgeons regarding the pressure threshold for the diagnosis of ACS and subsequent need for fasciotomy. The most effective method of diagnosis of ACS remains a clinical diagnosis requiring a high level of suspicion by the treating physician. Given the short duration of our patient’s procedure and lack of risk factors with the exception of lithotomy position and her full recovery after immediate recognition and surgical management, our case highlights the imperative of understanding the pathophysiology of ACS in lithotomy position and raising a high level of suspicion in a patient with postoperative calf pain after gynecological procedures.

## Conclusion

ACS is a rare but serious complication from surgical procedures performed in the lithotomy or hemilithotomy position. The greatest risk factor for major sequelae from ACS is failure to recognize and manage this condition in a timely fashion when a postoperative patient presents with increasing leg pain after surgery. Recommendations to prevent intraoperative development of a compartment syndrome include correct patient positioning [1, 23], utilizing well-padded stirrups for the lower extremity, to limit the duration in lithotomy position whenever possible, and to reposition and mobilize the knees and legs if procedures extend beyond 2 h of surgical time.

## Abbreviations

ACS, acute compartment syndrome; CK, creatinine kinase
